# A new use for an old index: preoperative high-density lipoprotein predicts recurrence in patients with hepatocellular carcinoma after curative resections

**DOI:** 10.1186/s12944-017-0509-3

**Published:** 2017-06-26

**Authors:** Lu Tian, Qian Yu, Xing-Hui Gao, Jiong Wu, Xiao-Lu Ma, Qian Dai, Chun-Yan Zhang, Yan Zhou, Yi-Chi Zhang, Bai-Shen Pan, Jian Zhou, Jia Fan, Xin-Rong Yang, Wei Guo

**Affiliations:** 1 Department of Laboratory Medicine, Zhongshan Hospital, Fudan University, 136 Yi Xue Yuan Road, Shanghai, 200032 People’s Republic of China; 2 Department of Liver Surgery, Liver Cancer Institute, Zhongshan hospital, Fudan University, Key Laboratory of Carcinogenesis and Cancer Invasion, Ministry of Education, 136 Yi Xue Yuan Road, Shanghai, 200032 People’s Republic of China

**Keywords:** HDL, HCC, Lipid metabolites, Prognosis

## Abstract

**Background:**

Hepatocellular carcinoma has high incidence and mortality worldwide. Liver is the site of most metabolic biotransformation, which could reflect the status of cells. Most plasma apolipoproteins, endogenous lipids and lipoproteins are synthesized in the liver. Therefore, the effects of lipid metabolites on prognosis of HCC deserved to be explored.

**Methods:**

We prospectively included 58 healthy donors (HD), 50 chronic hepatitis (CH) patients and a training cohort of 189 patients with HCC who underwent curative resections at Zhongshan Hospital from January 2012 to August 2012. We identified the optimal HDL_PO_ cutoff value at 0.98 mmol/L and used it to stratify patients into low- or high-HDL_PO_ groups for the entire cohort and four low-recurrent-risk subgroups. We also included an independent validation group of 182 HCC patients to validate this cutoff value. Prognostic values of HDL_PO_ and other factors were determined by Kaplan–Meier curves and the Cox proportional hazards model.

**Results:**

The low-HDL_PO_ group had a higher median tumor grade (*P* = 0.020) and a higher recurrence rate (*P* = 0.032). Results of multivariate analysis showed that preoperative γ-glutamyl transpeptidase (GGT) and HDL_PO_ were independent predictors of recurrence. Moreover, the predictive value of HDL_PO_ was retained in four low-recurrent-risk subgroups. As expected, clinicopathologic characteristics and predictive values were similar in the validation and training cohorts.

**Conclusions:**

HDL_PO_ is an accessible predictor of HCC recurrence after liver resections that can help identify patients who need more careful monitoring and follow-up care.

**Electronic supplementary material:**

The online version of this article (doi:10.1186/s12944-017-0509-3) contains supplementary material, which is available to authorized users.

## Background

Hepatocellular carcinoma (HCC) has third highest cancer mortality worldwide [1, 2]. Although advances in treatment have improved survival of patients with HCC, they suffer high post-surgical recurrence and metastasis rates [1, 3]. Despite the common use of alfa-fetoprotein (AFP) to diagnose and predict recurrence of HCC, its positive predictive rate is about 70% [4]. Therefore, identifying reliable indicators for patients with a high risk of post-surgical relapse is imperative to provide patients with optimal adjuvant therapy.

Metabolites are the best molecular indicators of cell status, and liver is the site of most metabolic biotransformation [5]. Most plasma apolipoproteins, endogenous lipids and lipoproteins are synthesized in the liver [6, 7]. Although high-density lipoprotein (HDL), as a lipid metabolite, is known as “good cholesterol,” with the main function of reverse cholesterol transport (RCT) and negative correlation with atherosclerosis [8], little is known about alterations of metabolism, especially HDL, and how these multi-level variations affect aggressive diseases and poor outcomes. Patients with hepatitis B or hepatitis C have been shown to have possible lipid disorders, including decreased plasma HDL [5, 9]. However, whether HDL levels are correlated with HCC prognosis is unclear. We therefore designed this single-center prospective study of 189 patients in the training group and 182 patients in the validation group, as well as 58 HD and 50 CH patients, to explore whether HDL levels could predict HCC recurrence.

## Methods

### Patients and specimens

We included a training cohort of 189 patients and a validation cohort of 182 patients who had undergone curative resections at our hospital from January 2012 to September 2013 for HCC, but had not yet accepted any radiotherapy or chemotherapy before then. The method of diagnosing HCC in these patients, and the inclusion and exclusion criteria, were described in a previous study [10]. We have added the inclusion and exclusion criteria in the Method. The inclusion criteria were: (1) definitive pathological diagnosis of HCC based on World Health Organization criteria, (2) curative resection, defined as complete macroscopic removal of the tumor, and (3) no prior anticancer treatment; the exclusion criteria were: (1) with other malignant tumors before operation; (2) with preoperative infection; (3) with blood and immune system diseases. Peripheral venous blood samples were collected before surgery, from which serum was separated by centrifuge and saved in a − 80 °C refrigerator. Tumor differentiation was graded by the Edmondson system, and staging was determined by the Barcelona Clinic Liver Cancer (BCLC) classifications [11]. The Zhongshan Hospital Research Ethics Committee approved this study, and all patients granted written informed consent.

### Follow-up

Patients’ postoperative surveillance included routine clinical and laboratory examinations, and imaging methods conducted within 1–3 months after their surgeries, to detect metastasis or recurrence [12]. Patients’ baseline clinical characteristics were noted, including sex, age, tumor number, size, encapsulation and grade, satellite lesions, vascular invasion, Child–Pugh grade, BCLC stage, AFP, alanine aminotransferase (ALT), γ-glutamyl transpeptidase (GGT), hepatitis virus B antigen (HBsAg), HDL, and any recurrences. All recipients were followed regularly until recurrence, death, or termination of the study. Time to recurrence (TTR) was defined as the period between surgery and recurrence.

### RNA isolation and RCR

Total RNA was isolated using TRIzol® LS reagent (Invitrogen) according to manufacturer’s instructions. The final elution volume ranged from 20 to 50 μl. The amount of specific transcripts was measured by RT-PCR using the 7500 quantitative Real-time PCR Machine.

### Statistical analysis

Statistical analysis was used SPSS for Windows, Version 16.0 (SPSS Inc., Chicago, IL, USA). Student *t* test, Pearson’s χ^2^ test, and Fisher’s exact test were used to compare differences between two groups. Univariate and multivariate analyses used the Cox proportional hazards regression model. The Kaplan–Meier method was used to generate survival curves, and differences between groups were assessed using the log-rank test. A two-sided *P* value <0.05 was considered significant. The X-tile 3.6.1 software (Yale University, New Haven, CT) was used for bioinformatic analysis of the cohort data to determine the cutoff value of preoperative HDL (HDL_PO_) for tumor recurrence [13].

## Results

### Lipid metabolism screening and optimal HDL_PO_ cutoff

**Fig. 1:**
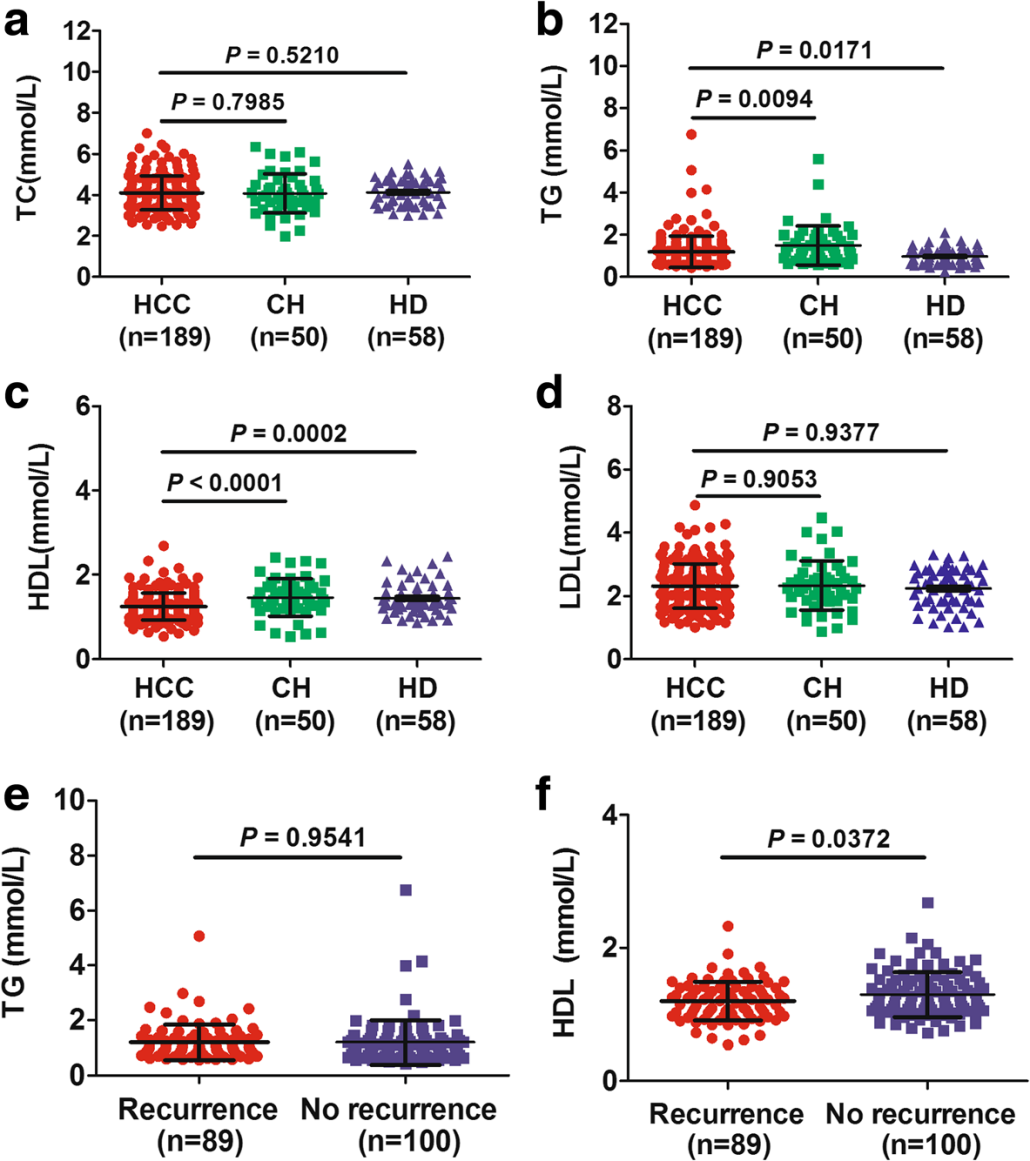
Lipid metabolites in different groups. Levels of total cholesterol (TC; **a**), triglycerides (TG; **b**), high-density lipoprotein (HDL; **c**) and low-density lipoprotein (LDL; **d**) in HCC patients,chronic hepatitis (CH) patients, and healthy donors (HD); and TG (**e**) as well as HDL (**f**) were analyzed in patients who suffered recurrences and those whose HCC did not recur

We examined four products of lipid metabolism, including total cholesterol (TC), triglycerides (TG), HDL, and low-density lipoprotein (LDL) in 189 HCC patients before surgery, 58 healthy donors (HD) and 50 chronic hepatitis(CH) patients. As expected, HDL_PO_ was significantly lower in HCC patients than CH patients (*P* < 0.001) and HD (*P* = 0.0002), whereas TG was significantly lower than CH patients (*P* = 0.0094) and higher than HD (*P* = 0.0171; Fig. 1 a-d). We then focused our research on these two markers to explore their differences in groups of patients who did, and who did not, suffer recurrences. We found only HDL_PO_ clearly discriminated between the two groups (*P* = 0.037; Fig. 1 e-f).

Results from X-Tile analysis revealed an optimal cutoff point for HDL_PO_ at 0.98 mmol/L among the training cohort patients (Additional file 1: Figure S1), who were then stratified into those with ≤ 0.98 mmol/L (low HDL_PO_) or >0.98 mmol/L (high HDL_PO_) for all subsequent analyses. The cutoff point generated by X-tile was then verified in an independent validation cohort to extend the universal use of 0.98 mmol/L.

### Correlations between clinicopathologic characteristics and HDL_PO_ levels in the training cohort

The 189 patients included 162 men (85.71%) and 27 women (14.29%) with a median age of 54.4 years (range: 37–87 years). We found 166 (87.83%) to be HBsAg-positive. The recurrence rate for all patients was 47.62% by the end of the follow-up (median TTR: 30.9 months; range: 0.6–37 months). After applying the 0.98 mmol/L HDL_PO_ cutoff, 32 patients (16.93%) were in the low-HDL_PO_ group and 157 (83.07%) in the high-HDL_PO_ group.

Clinicopathologic characteristics included sex, age, tumor number and size, encapsulation and grade, satellite lesions, vascular invasion, Child–Pugh grade, BCLC stage, AFP, ALT, GGT, HBsAg, and recurrence. The low-HDL_PO_ group had more advanced median tumor grades (*P* = 0.020) and a higher recurrence rate (*P* = 0.032, Table 1).

**Table 1:** The correlation between clinicopathologic characteristics and HDL_PO_ in the training cohort

		Number (*n* = 189)	Low-HDL_PO_ group (*n* = 32)	High-HDL_PO_ group (*n* = 157)	*P* value
Sex	Male	163	28	135	0.821^a^
	Female	26	4	22	
Age	≤50	69	13	56	0.596
	>50	120	19	101	
Tumor number	Single	151	26	125	0.833
	Multiple	38	6	32	
Tumor size	≤5	120	18	102	0.351
	>5	69	14	55	
Tumor encapsulation	None	115	17	98	0.326
	Complete	74	15	59	
Satellite lesions	No	166	27	139	0.524^a^
	Yes	23	5	18	
Vascular invasion	No	118	17	101	0.321
	Yes	71	15	56	
Tumor grade	I-II	117	14	103	0.020
	III-IV	72	18	54	
Child-Pugh grade	A	178	29	149	0.347^a^
	B	11	3	8	
BCLC stage	0 + A	138	24	114	0.781
	B + C	51	8	43	
AFP,μg/L	≤400	139	21	118	0.265
	>400	50	11	39	
ALT,μg/L	≤40	178	32	146	0.124^a^
	>40	11	0	11	
GGT,IU/L	≤54	110	17	93	0.559
	>54	79	15	64	
HbsAg	Negative	23	1	22	0.100^a^
	Positive	166	31	135	
Recurrence	No	100	11	89	0.032
	Yes	89	21	68	

Abbreviations: AFP, α-fetoprotein; ALT, alanine aminotransferase; GGT, γ-glutamyl transpeptadase; HBsAg, hepatitis B surface antigen; BCLC, Barcelona Clinic Liver Cancer ^a^Fisher’s exact test

### The prognostic value of HDL_PO_ level for HCC patients in the training cohort

The Cox proportional hazard model was used to determine prognostic indicators. In univariate analysis, discriminating variables were preoperative GGT (hazard ratio [HR]: 1.004; 95% confidence index [95% CI]: 1.002–1.005; *P* = 0.001) and HDL_PO_ (HR: 0.526; 95% CI: 0.322–0.858; *P* = 0.010). These two variables were further evaluated in multivariate analysis, which showed both GGT (HR, 1.986; 95% CI, 1.308–3.017; *P* = 0.001) and HDL_PO_ (HR: 0.519; 95% CI: 0.318–0.848; *P* = 0.009) to be independent prognostic risk factors of HCC recurrence (Table 2).

**Table 2:** Univariate and multivariate Cox proportional hazard analysis of factors associated with recurrence in the training cohort

	Univariate analysis		Multivariate analysis	
	HR(95% CI)	*P* value	HR(95% CI)	*P* value
Sex	1.130 (0.601–2.124)	0.705	NA	NA
Age	0.940 (0.612–1.446)	0.780	NA	NA
Tumor number	0.910(0.536–1.543)	0.725	NA	NA
Tumor size	1.025(0.667–1.576)	0.910	NA	NA
Tumor encapsulation	1.115(0.727–1.710)	0.617	NA	NA
Satellite lesion	1.090(0.580–2.049)	0.789	NA	NA
Vascular invasion	0.899(0.575–1.375)	0.596	NA	NA
Tumor grade	1.092(0.714–1.671)	0.685	NA	NA
Child-Pugh grade	1.785(0.824–3.865)	0.142	NA	NA
BCLC stage	0.606(0.361–1.017)	0.058	NA	NA
AFP	0.906 (0.563–1.456)	0.682	NA	NA
ALT	0.627(0.230–1.709)	0.362	NA	NA
GGT	1.004(1.002–1.005)	0.001	1.986(1.308–3.017)	0.001
HbsAg	1.612(0.780–3.333)	0.198	NA	NA
HDL_PO_	0.526(0.322–0.858)	0.010	0.519(0.318–0.848)	0.009

Note:HR is hazard ratio Abbreviation: NA,not applicable

**Fig. 2:**
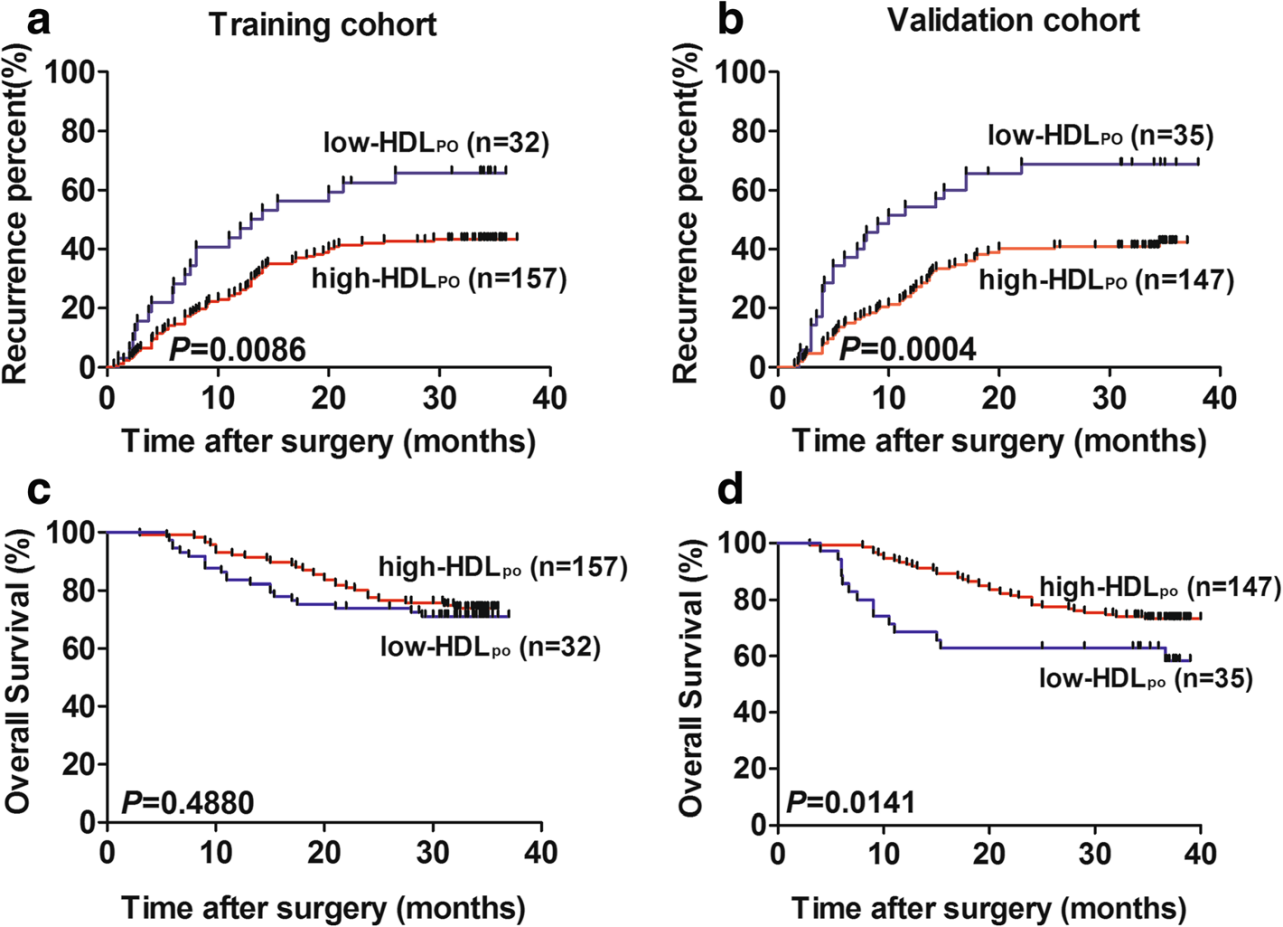
Prognostic significance of HDL_PO_ in the training and validation cohorts. Kaplan–Meier analysis for time to recurrence (TTR) and overall survival (OS) for patients with low- vs high-HDL_PO_ in the training cohort (**a**, **c**) and the validation group (**b**, **d**)

In the training cohort, Kaplan–Meier analysis showed the low- and high-HDL_PO_ groups to significantly differ in median TTR (low-HDL_PO_ group: 13.5 months; high-HDL_PO_ group: 31.1 months; *P* = 0.0086 Fig. 2a). The low-HDL_PO_ group had a significantly higher recurrence rate (65.63%) than the high-HDL_PO_ group (43.31%). However, there were no significant statistics difference in OS between the two groups (*P* = 0.4880 Fig. 2c).

**Fig. 3:**
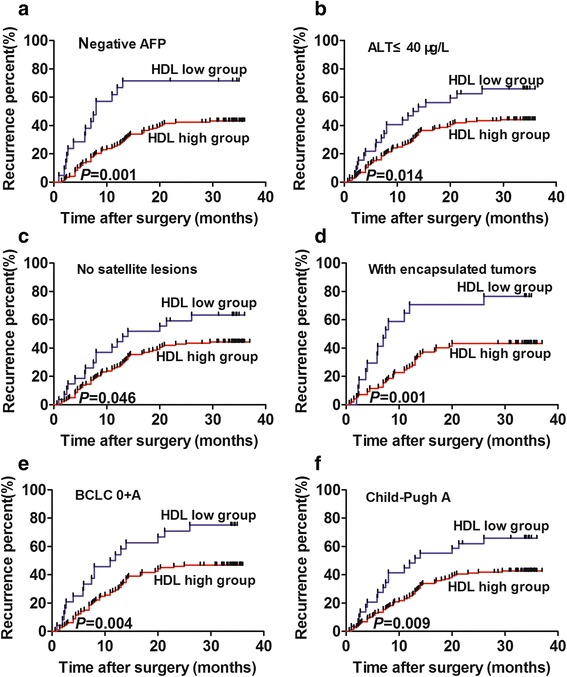
The predictive value of HDLPO shown in 4 low-risk subgroups in the training cohort. Kaplan–Meier analysis of TTR for HCC patients with negative AFP levels (**a**), ALT≤40 μg/L (**b**), with no satellite lesions (**c**), with encapsulated tumors (**d**), with BCLC stage 0 + A disease (**e**), and Child-Pugh A (**f**).

Further analysis in HCC patients with AFP negative or other low recurrent risks among the training cohort, we found that HDL_PO_ level retained significant prognostic value in patients who were AFP-negative (*P* = 0.001), with no satellite lesions (*P* = 0.046), with complete encapsulated tumors (*P* = 0.001), and with BCLC stage 0 + A disease (*P* = 0.004; Fig. 3 a-e). The recurrence rates of each subgroup were listed in Additional file 2 **:** Table S1.

### Verifying the HDL_PO_ cutoff point and its correlation with clinicopathologic characteristics in the validation cohort

The cutoff value was defined using X-tile software. To dispel possible bias from this particular cohort, we used an independent validation cohort to verify the cutoff value. As expected, Kaplan–Meier analysis showed the low- and high-HDL_PO_ groups to significantly differ in median TTR (low-HDL_PO_ group: 10.0 months; high-HDL_PO_ group: 32.2 months; *P* = 0.0004 Fig. 2b) and OS(*P* = 0.0141 Fig. 2d). The low-HDL_PO_ group also had a significantly higher recurrence rate (68.57%) than did the high-HDL_PO_ group (41.50%).

We also compared the clinicopathologic characteristics of the validation cohort with those of the training cohort, and found that they were similar in sex, age, and other characteristics (Table 3), as was the recurrence rate (46.70% by the end of the follow-up; median TTR: 30.9 months; range: 1.0–38.0 months). The low-HDL_PO_ group had more advanced median tumor grades (*P* = 0.032) and a higher recurrence rate (*P* = 0.005).

**Table 3:** The correlation between clinicopathologic characteristics and HDL_PO_ in the validation cohort

		Number (*n* = 182)	Low-HDL_PO_ group (*n* = 35)	High-HDL_PO_ group (*n* = 147)	*P* value
Sex	Male	158	32	126	0.371^a^
	Female	24	3	21	
Age	≤50	64	13	51	0.845
	>50	118	22	96	
Tumor number	Single	144	27	117	0.817
	Multiple	38	8	30	
Tumor size	≤5	115	19	96	0.246
	>5	67	16	51	
Tumor encapsulation	None	112	19	93	0.340
	Complete	70	16	54	
Satellite lesions	No	160	30	130	0.663^a^
	Yes	22	5	17	
Vascular invasion	No	112	19	93	0.340
	Yes	70	16	54	
Tumor grade	I-II	114	16	98	0.032
	III-IV	68	19	49	
Child-Pugh grade	A	171	31	140	0.138^a^
	B	11	4	7	
BCLC stage	0 + A	130	25	105	0.575
	B + C	52	10	42	
AFP,μg/L	≤400	135	22	113	0.131
	>400	47	13	34	
ALT,μg/L	≤40	170	34	136	0.278^a^
	>40	12	1	11	
GGT,IU/L	≤54	101	19	82	1.000
	>54	81	16	65	
HbsAg	Negative	23	1	22	0.053^a^
	Positive	159	34	125	
Recurrence	No	97	11	86	0.005
	Yes	85	24	61	

Abbreviations: AFP, α-fetoprotein; ALT, alanine aminotransferase; GGT, γ-glutamyl transpeptadase; HBsAg, hepatitis B surface antigen; BCLC, Barcelona Clinic Liver Cancer ^a^Fisher’s exact test

Finally, univariate analysis of the validation group showed that Child-Pugh score (HR: 2.601; 95% CI: 1.252–5.402; *P* = 0.010), GGT (HR:1.826; 95% CI: 1.193–2.795; *P* = 0.006) and HDL_PO_ (HR: 0.436; 95% CI: 0.271–0.700; *P* = 0.001) to be significantly associated with recurrence. In multivariate analysis, all of these variables independently predicted HCC recurrence (Child-Pugh score, HR: 2.358; 95% CI: 1.125–4.945; *P* = 0.023; GGT, HR: 1.863; 95% CI: 1.216–2.853; *P* = 0.004; and HDL_PO_, HR: 0.448; 95% CI: 0.277–0.723; *P* = 0.001; Additional file 2: Table S2).

### The sourse and metabolism of HDL

**Fig. 4:**
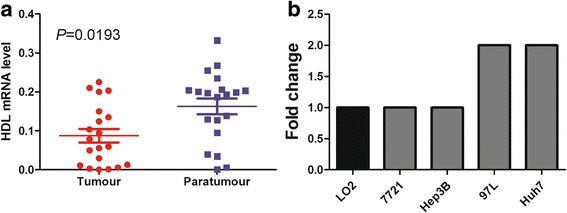
The sourse and metabolism of HDL. The HDL mRNA level of tumor tissue and paratumor tissue (**a**); HDL concentration of HCC cell lines and normal live cell line (**b**)

To explore the mechanism of low HDL_PO_ with poor prognosis, we detected HDL mRNA level of tumor and paratumor tissues from 20 patients. The results showed that paratumor tissue presented higher level of HDL than tumor tissue (*P* = 0.0193, Fig. 4a). Moreover, the cell surpernatant from high invasive HCC cell lines presented lower HDL level than normal live cell line and low invasive HCC cell lines (4b).

## Discussion

A meta-analysis of 24 large randomized controlled trials of lipid-modifying therapy showed an inverse relationship between HDL and risk of cancer [14]. Fan Y, et al. demonstrated that in the triple-negative breast cancer group, patients with low HDL suffered worse relapse-free survival (RFS) and overall survival (OS), and low HDL was an independent worse prognostic factor for both RFS and OS [15]. Moreover, other studies had validated HDL could be used as an indicator in, lung cancer [16], prostate cancer [17], and colorectal cancer [18]. Cancer is known to be a proinflammatory state, in which inflammatory cells actively participate in the neoplastic process, allowing tumor cell proliferation, migration, and survival [19, 20]. The relationship between inflammation and lipid metabolism has become a focus of investigation. As a metabolite, HDL plays an extremely important role in protecting the cardiovascular system from atherosclerosis, by mediating cholesterol transport. Plausibly, HDL protects against cancer development through its pleiotropic properties, including anti-oxidation and modulating cytokine production, and by blocking apoptosis, cell-growth stimulation and migration [16, 21, 22].

Although several studies have found clues to the relationships between HDL and hepatitis or HCC [5, 23–25], we are the first to predict prognoses of HCC patients by their HDL_PO_ levels in both training and validation groups, and to extend this predictive value to low-recurrent-risk subgroups, thereby strengthening the clinical utility of this commonly accessible test to predict HCC recurrence. As serum HDL is analyzed routinely in clinical laboratories, detection of HDL_PO_ can easily be standardized for early decision-making to tailor the most effective therapy for each HCC patients.

More profoundly, we found that HDL_PO_ was also a potent indicator for patients in four low-recurrent-risk subgroups, in whom recurrences were not easily detected by routine blood tests and pathologic results. AFP is commonly used to diagnose and monitor HCC patients’ conditions after surgery [26], but its role in early dignosis and predicting prognosis of HCC were still in debate, especially some HCC patients were still AFP-negative, so AFP still had some limitations as an screening or predictive indicator. Satellite lesions indicate the cancer is not confined to one site and has disseminated in the liver [27]; encapsulation help limit the tumor in a separate enclosure and prevent it from spreading [28]; BCLC stage 0 + A [29] is common early HCC assessments. These factors were considered to be recurrence related, and the negative condition of them had low indices of clinical suspicion. Physicians should consider HDL_PO_ as a stratification factor when making management decisions for HCC patients.

The progression of HCC is a complex, multi-step process, and disordered lipid metabolism might contribute to poor prognosis. It was reported that lipid oxidation and resulting oxidized lipid mediated inflammation appear to be common to the etiology of a number of inflammatory diseases [30, 31], implicating a role for lipoproteins in the development and metastasis of cancer. Since HDL was mainly formed in the liver, and our results demonstrated that HDL produced by hepatoma cells was less than normal liver cells. Jessica Fioravanti, et al. reported that after incubation of woodchuck WCH17 cell line with DiI-labled HDL, the cell line internalized the fluorescent HDL, which verified HDL could uptake by hepatoma cells [32]. HDL, as a structural component of the cell membrane and is localized in membrane microdomains that assemble the signal transduction machinery and associate to proteins implicated in key cellular signaling pathways, played the protective role from metastasis through enhancing anti-inflammatory and anti-oxidant properties [33]. Because this indicator is a promising means of evaluating prognosis of patients with HCC, we strongly recommend that doctors consider HDL_PO_ when making management decisions. For instance, patients with lower HDL_PO_ should be provided with earlier and more frequent imaging or other tests to detect micro-recurrence lesions.

Our study is limited by the fact that the HDL levels were measured only once at baseline before surgery, and do not reflect random fluctuations over time, which would tend to increase the data variance. Moreover, we focused only on preoperative HDL levels without making notice of postoperative values, which confined the clinical utility of this index. As HCC patients in China have a high positive rate for HBsAg, ethnic differences in lipoprotein levels should be considered, and HDL_PO_ cutoff values should be redefined when generalizing the findings to persons with different ethnicities.

To our knowledge, this is the first report to predict prognosis of patients with HCC though HDL_PO_ levels. Although the underlying mechanism is unclear, the results validate HDL_PO_ as an assistant predictor of post-surgical recurrence in HCC patients.

## Conclusions

In summary, this is the first report showing a novel use of HDL in predicting the prognosis of HCC. We compared four lipid metabolites in healthy donors, chronic hepatitis patients and HCC patients and found the distinctive value of HDL. As expected, low-HDL_PO_ group had a higher median tumor grade and were more likely to suffer recurrence. Moreover, the predictive value of HDL_PO_ was retained in four low-recurrent-risk subgroups. HDL_PO_ could be a accessible predictor of HCC recurrence after liver resections and help identify patients who need more careful monitoring and follow-up care.

## Additional files

Additional file 1: Figure S1. The cutoff point generated by X-tile. (TIFF 1880 kb)

Additional file 2: Table S1. The summary of four subgroups. **Table S2.** Univariate and multivariate Cox proportional hazard analysis of factors associated with recurrence in the validation cohort. (DOCX 14 kb)

## Abbreviations

AFP: α-fetoprotein
ALT: alanine aminotransferase
BCLC: Barcelona Clinic Liver Cancer
CH: chronic hepatitis
GGT: γ-glutamyl transpeptidase
HBsAg: hepatitis virus B antigen
HCC: hepatocellular carcinoma
HD: healthy donors
HDL: high-density lipoprotein
HDL_PO_: preoperative high-density lipoprotein
HR: hazard ratio
LDL: low-density lipoprotein
NA: not applicable
RCT: reverse cholesterol transport
TC: total cholesterol
TG: triglycerides
TTR: time to recurrence
