# Supplementary figures and images for: A new use for an old index: preoperative high-density lipoprotein predicts recurrence in patients with hepatocellular carcinoma after curative resections
Source: Lipids Health Dis. 2017 Jun 26;16:123. doi: 10.1186/s12944-017-0509-3 (PMC5485717; doi:10.1186/s12944-017-0509-3)

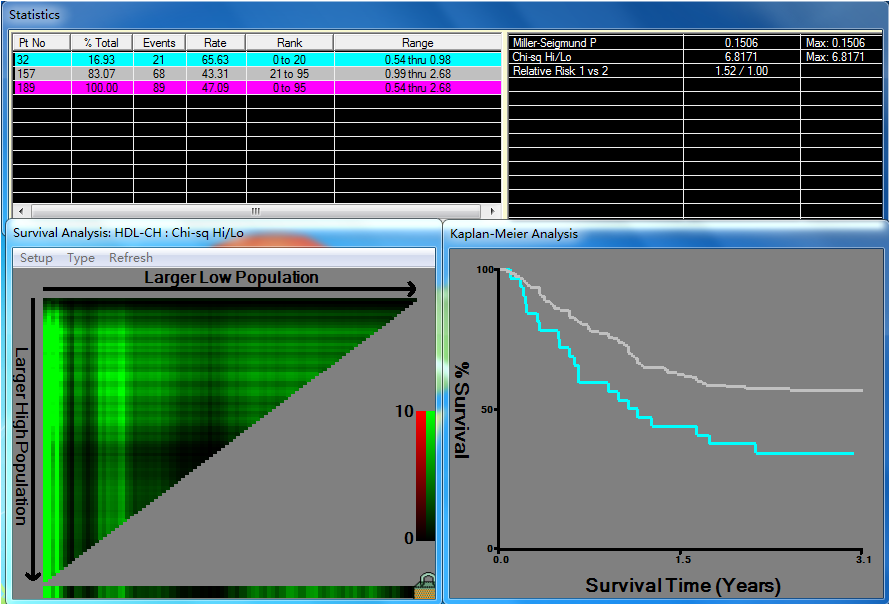

Supplement: Supplementary file 1 — The cutoff point generated by X-tile. (TIFF 1880 kb) [file 12944_2017_509_MOESM1_ESM.tif]
