# Supplementary material for: A new use for an old index: preoperative high-density lipoprotein predicts recurrence in patients with hepatocellular carcinoma after curative resections
Source: Lipids Health Dis. 2017 Jun 26;16:123. doi: 10.1186/s12944-017-0509-3 (PMC5485717; doi:10.1186/s12944-017-0509-3)
Supplement: Supplementary file 2 — The summary of four subgroups. Table S2. Univariate and multivariate Cox proportional hazard analysis of factors associated with recurrence in the validation cohort. (DOCX 14 kb) [file 12944_2017_509_MOESM2_ESM.docx]

| **Table S1 The summary of four subgroups** | | | |  |  |
| --- | --- | --- | --- | --- | --- |
|  | | Recurrence Rate | | P value | |
|  | | Low-HDL_PO_ group | High-HDL_PO_ group |  |  |
| AFP-negative | | 71.43% | 43.22% | 0.001 | |
| No satellite lesions | | 62.96% | 43.88% | 0.046 | |
| Encapsulated tumors | | 76.47% | 42.86% | 0.001 | |
| BCLC stage 0+A | | 75.00% | 46.90% | 0.004 | |

| **Table S2 Univariate and multivariate Cox proportional hazard analysis of factors associated with recurrence in the validation cohort** | | | | |
| --- | --- | --- | --- | --- |
|  | Univariate analysis | | Multivariate analysis | |
|  | HR(95% CI) | P value | HR(95% CI) | P value |
| Sex | 1.235 (0.619-2.464) | 0.550 | NA | NA |
| Age | 0.885 (0.569-1.376) | 0.587 | NA | NA |
| Tumor number | 0.950(0.558-1.616) | 0.849 | NA | NA |
| Tumor size | 1.135(0.734-1.756) | 0.569 | NA | NA |
| Tumor encapsulation | 1.119(0.721-1.736) | 0.616 | NA | NA |
| Satellite lesion | 1.027(0.531-1.986) | 0.938 | NA | NA |
| Vascular invasion | 0.975(0.629-1.513) | 0.911 | NA | NA |
| Tumor grade | 1.074(0.694-1.662) | 0.747 | NA | NA |
| Child-Pugh score | 2.601(1.252-5.402) | 0.010 | 2.358(1.125-4.945) | 0.023 |
| BCLC stage | 0.662(0.397-1.104) | 0.114 | NA | NA |
| AFP | 0.888 (0.543-1.455) | 0.638 | NA | NA |
| ALT | 0.791(0.320-1.953) | 0.611 | NA | NA |
| GGT | 1.826(1.193-2.795) | 0.006 | 1.863(1.216-2.853) | 0.004 |
| HbsAg | 1.567(0.757-3.247) | 0.226 | NA | NA |
| HDL_PO_ | 0.436(0.271-0.700) | 0.001 | 0.448(0.277-0.723) | 0.001 |
| Note ：HR is hazard ratio Abbreviation: NA,not applicable. | | | | |
